# Common Variations in Prothrombotic Genes and Susceptibility to Ischemic Stroke in Young Patients: A Case-Control Study in Southeast Iran

**DOI:** 10.3390/medicina55020047

**Published:** 2019-02-13

**Authors:** Seyed Mehdi Hashemi, Nourollah Ramroodi, Hamed Amiri Fard, Sahar Talebian, Maryam Haghighi Rohani, Mahnaz Rezaei, Mehrangiz Noora, Saeedeh Salimi

**Affiliations:** 1 Clinical Immunology Research Center, Ali-ebne Abitaleb Hospital, Zahedan University of Medical Sciences, Zahedan 9816743111, Iran; mehdihashemi3107@gmail.com; 2 Department of Internal Medicine, School of Medicine, Zahedan University of Medical Sciences, Zahedan 9816743111, Iran; 3 Department of Neurology, School of Medicine, Zahedan University of Medical Sciences, Zahedan 9816743111, Iran; ramrodin@zaums.ac.ir (N.R.); dr.amirifard@gmail.com (H.A.F.); 4 Cancer Research Center, Mashhad University of Medical Sciences, Mashhad 91388 13944, Iran; sahartalebian39@yahoo.com; 5 School of Medicine, Zahedan University of Medical Sciences, Zahedan 9816743175, Iran; M.rohani72@gmail.com; 6 Department of Clinical Biochemistry, School of Medicine, Zahedan University of Medical Sciences, Zahedan 9816743175, Iran; mrezaei550@gmail.com (M.R.); naslelm1@gmail.com (M.N.); 7 Cellular and Molecular Research Center, Zahedan University of Medical Sciences, Zahedan 9816743111, Iran

**Keywords:** ischemic stroke, Factor V, *MTHFR*, prothrombin, polymorphism

## Abstract

*Background and Objective:* Evidence indicates that genetic factors may be involved in the risk of ischemic stroke (IS). The aim of this study was to assess the effect of genetic polymorphisms located in exons or untranslated regions of *MTHFR* as well as *FV* genes on ischemic stroke. *Materials and Methods:* In this case-control study, 106 patients with IS and 157 healthy volunteers (age <50 years) were genotyped for *MTHFR* C677T, *A1298C*, *C2572A* and *C4869G*, *FVL*, and prothrombin *G20210A* polymorphisms. *Results:* The *MTHFR* 677CT genotype was more frequent in patients and increased risk of IS with Odds Ratio = 1.9. The *MTHFR* A1298C and *C2572A* polymorphisms were not associated with IS in dominant and recessive models. Our findings showed a significant decrease in the *MTHFR* *4869CG* genotype in IS patients, and this variant was associated with a decreased risk of IS in the dominant model. The *CAAT* haplotype was associated with increased risk, and the *GAAC* haplotype was associated with decreased risk of IS compared to other haplotypes. There was no relation between *FVL* G1691A polymorphism and IS risk. *Conclusions:* The present study showed that the *MTHFR* 677CT genotype was more frequent and the *MTHFR* 4869CG genotype was less frequent in young IS patients.

## 1. Intoduction

Stroke is a set of devastating and debilitating diseases that are the result of blockage to blood vessels in the brain. Stroke is the third debilitating and second deadliest disease in the world, and is responsible for about 10% of all deaths. Five and a half million people die annually from ischemic stroke (IS), and the resulting disability-adjusted life years (DALYs) loss was equivalent to US $44 million in 2011 [1,2]. Released statistics show that low- and middle-income countries account for 70% of strokes and 87% of stroke-related deaths—and its incidence rate has doubled in these countries over the past four decades [3]. Commonly, the mean age of stroke patients is 15 years younger and the mortality rate of stroke patients is higher in countries with low and middle income compared to countries with high income [4]. The World Health Organization (WHO) indicated stroke as the second greatest cause of mortality in Iran, killing 41,600 people in 2012, equivalent to 10.5% of all deaths [5]. Stroke mostly occurs at peak productive age. Despite its massive impact on the socio-economic development of countries, this growing crisis has received very slight attention so far. Stroke is largely preventable and treatable, and is an avoidable tragedy for patients, families, and societies. Stroke can be divided into two major types: brain ischemia (due to thrombosis, embolism, systemic hypo-perfusion or blood disorders) and brain hemorrhage [6]

IS represents approximately 10% of all cerebrovascular diseases. The exact etiology of IS is complicated; it has numerous risk factors such as diabetes mellitus, hypertension, and age. Also, the complexity of genetic and environmental factors could be involved [7]. It is believed that, similar to other vascular complications, genetic variants play a key role in IS susceptibility [8,9,10].

Thrombophilia is a widespread range of changes that promote conditions for vascular thrombosis due to hematological changes that induce blood hypercoagulability; these can be inherited or acquired [11,12]. Among the inherited factors of thrombophilia, the four most common genetic markers are Factor V Leiden (*FVL*, G1691A), methylenetetrahydrofolate reductase polymorphisms (*MTHFR C677T* and *A1298C)*, prothrombin gene (*FII*, *G20210A*), and plasminogen activator inhibitor-1 (*PAI-1*) variants—and their relation to various vascular diseases is known [13].

**Figure 1:**
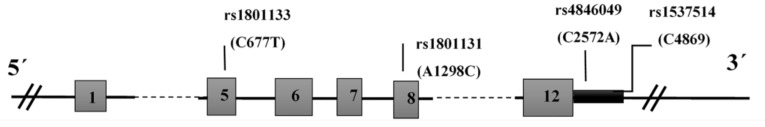
The schematic diagram of *MTHFR* polymorphisms.

Previous studies reported an association between elevated levels of homocysteine—which is an endothelial toxin—and the risk of vascular events, including IS [14]. Evidence showed that the 677TT and 1298CC genotypes (mutant homozygous) of the *MTHFR* gene are associated with hyperhomocysteinemia (HHcy) and have a critical role in modulating plasma homocysteine concentrations—which predispose the carrier to thrombosis and IS [15,16]. The effects of the *MTHFR* C677T polymorphism were highest in the regions where the diet has a low folate content [17]. Regarding *FVL*, the substitution of arginine by glutamine at 506 amino acid residue increases its resistance to degradation by activated protein C and accentuates the venous thromboembolism risk. At position 20210 of the 3′-UTR region of the Factor II gene, a G to A transition has been associated with higher levels of prothrombin and more risk for venous thrombosis [16]. Recently, the association between several polymorphisms in the 3′-UTR region of the *MTHFR* gene (Figure 1) and susceptibility to various diseases has been reported [16,18].

Although the exact mechanisms that lead to IS in young adults are not well understood, controversial studies in recent years showed evidence on the causality of IS in patients with methylenetetrahydrofolate reductase, prothrombin, and *FVL* gene polymorphisms. Therefore, in this study the possible effects of common *MTHFR C677T*, *A1298C*, *C2572A*, and *C4869G*, as well as *FVL* and prothrombin *G20210A* polymorphisms on IS in young patients were evaluated in a southeastern Iranian population.

## 2. Materials and Methods

### 2.1. Participants

This case-control study was carried out from May 2016 to September 2017 on 106 cases with IS and 157 control subjects who were matched to the IS patients for age and sex. All patients and controls were younger than 50 years in age. Informed consent was obtained from all participants, and the study was approved by the ethics committee of the Zahedan university of medical sciences (code 7185). The inclusion criteria for IS patients was the existence of IS at the present hospital admission, which the computerized tomography (CT) or magnetic resonance imaging (MRI) scan and clinical symptoms confirmed. The exclusion criteria were patients who had experienced an acute hemorrhagic stroke or other neurological diseases, a known malignancy, renal and liver disease, hematologic disorders, and hypothyroidism. Cases with IS of a cardioembolic origin (e.g., with atrial fibrillation) in both groups were also excluded.

The questionnaire was completed by skilled nurses to collect data on demographic, clinical, and lifestyle characteristics of patients and controls.

Blood pressure was measured for all subjects in the seated position with the back supported and legs uncrossed. Participants rested for at least 5 min before the assay. Blood pressure was measured using a routine mercury sphygmomanometer device (Omron HEM-711 IntelliSense, Tokyo, Japan). We repeated all assays twice and the average values were considered.

Smokers were known as those who smoked tobacco regularly up to six months prior with constant or intermittent usage (at least one cigarette per week).

### 2.2. Genomic DNA Extraction and Genotyping

Blood samples taken from the study subjects were drained into tubes containing Ethylenediaminetetraacetic acid (EDTA) and kept in a −20 °C freezer. To extract genomic DNA, the salting out method was performed on peripheral blood leukocytes. To analyze *MTHFR*, *FVL*, and prothrombin polymorphisms, the polymerase chain reaction-restriction fragment length polymorphism (PCR-RFLP) was performed as previously described [19,20].

### 2.3. Statistical Analysis

SPSS version 20 (SPSS Inc., Chicago, IL, USA) was employed to analyze the data. The Kolmogorov–Smirnov test was used to evaluate the normality of quantitative variables. The clinical data and demographic characteristics of the study groups were compared by the Fisher exact and student *t* tests. Logistic regression analyses, adjusted for age and gender, were employed to calculate the associations between genotypes and disease based on Odds Ratio (OR) and 95% Confidence Interval (CI). HaploView software was used to analyze the frequency and Linkage Disequilibrium (LD) of haplotypes. *p* < 0.05 was considered the level of significance.

## 3. Results

### 3.1. Demographic Characteristics

Table 1 presents the clinical and demographic characteristics of 106 patients with IS (<50 years), and 157 sex- and age-matched controls. There was no statistically significant difference in the systolic and diastolic blood pressures between the two groups. TG, total cholesterol, and LDL cholesterol levels were higher in the IS group, but the differences were not statistically significant.

### 3.2. MTHFR Polymorphisms and Ischemic Stroke Risk

The alleles and genotypes’ frequency of *MTHFR* polymorphisms in IS patients and the control group are shown in Table 2.

### 3.3. MTHFR C677T Polymorphism

The frequency of individuals carrying the *MTHFR 677 CT* genotype was statistically higher in the IS group, and this genotype was associated with a 1.9-fold greater risk of IS (OR 1.9 (95% CI 1.1–3.4); *p* = 0.028). The 677TT genotype was more frequent in IS patients, but not statistically different (OR 1.8 (95% CI 0.7–5); *p* = 0.26). Moreover, the *MTHFR C677T* polymorphism was associated with a higher risk of IS in dominant (OR 1.9 (95% CI 1.1–3.3); *p* = 0.019) but not recessive models (OR 1.5 (95% CI 0.6–4.2); *p* = 0.4).

### 3.4. MTHFR A1298C Polymorphism

The frequency of individuals carrying the *MTHFR* 1298AC and CC genotypes did not differ between the two groups, therefore this polymorphism was not associated with IS risk.

### 3.5. MTHFR C2572A Polymorphism

The frequencies of the *MTHFR* 2572AC and CC genotypes were not statistically different between patients and controls (*p* = 0.12 and *p* = 0.23 respectively), and this variant was not associated with IS in dominant and recessive models.

### 3.6. MTHFR C4869G Polymorphism

The frequency of the *MTHFR 4869CG* genotype was significantly reduced in the IS group, and this genotype was associated with decreased IS risk (OR 0.4 (95% CI 0.2–0.9); *p* = 0.016). In addition, *MTHFR C4869G* polymorphism was associated with lower risk of IS in the dominant (OR 0.4 (95% CI 0.2–0.8); *p* = 0.01) model.

### 3.7. Haplotype Analysis of MTHFR Polymorphisms

**Figure 2:**
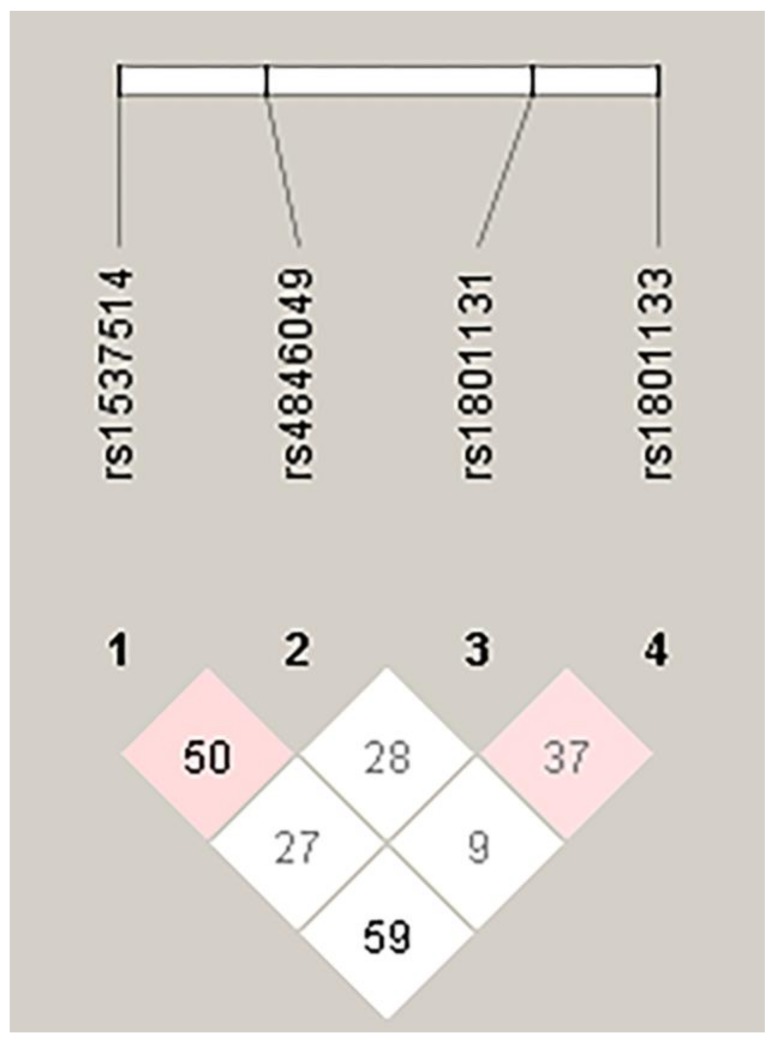
The linkage disequilibrium pattern of *MTHFR* polymorphisms.

Table 3 shows the results from the haplotype analysis of *MTHFR C4869G*, *C2572A*, *A1298C*, and *C677T* polymorphisms in IS and control groups. The CCAC haplotype was the most frequent haplotype in IS and control groups. The frequency of individuals carrying the CAAT haplotype was statistically higher in IS patients than controls (8.5 vs. 3.2%) compared to other haplotypes, and this haplotype was associated with a 2.8-fold higher risk of IS (OR 2.8 (95% CI 1.3–6.2); *p* = 0.01). However, the frequency of the GAAC haplotype was significantly lower in IS patients than controls (3.8 vs. 9.2%) compared to other haplotypes, and this haplotype could be a protective genetic factor against IS (OR 0.4 (95% CI 0.2–0.9); *p* = 0.02). Figure 2 shows the linkage disequilibrium between *MTHFR* polymorphisms in the total studied population.

### 3.8. FVL G1691A and Prothrombin G20210A Polymorphisms and Ischemic Stroke Risk

The frequency of the *FVL* 1691GA genotype was higher in IS patients; however, the difference was not significant (OR 2 (95% CI 0.9–4.3); *p* = 0.09). We did not observe the *FVL* 1691AA genotype in IS and control groups. Moreover, we did not find the prothrombin 20210A allele in our study population.

## 4. Discussion

Hyperhomocysteinemia (HHcy) is a pathological complication described by an increase in the plasma level of total homocysteine [21]. Numerous epidemiological investigations have shown the role of HHcy in premature atherosclerosis and thrombotic disease susceptibility. Evidence has revealed that HHcy leads to endothelial dysfunction and causes apoptotic cell death in various cells—including endothelial ones [22,23]. HHcy is also known to be associated with improved thrombotic disorders and has been considered a risk factor for atherosclerosis and thrombosis—therefore, the elevated levels of plasma homocysteine have been related with susceptibility to IS, coronary artery disease (CAD), and pregnancy hypertension [24,25,26].

Evidence has shown that elevated homocysteine levels could be due to genetic defects in enzymes involved in its metabolism, or environmental factors such as vitamin B12 or folic acid deficiency [27]. *MTHFR* plays an important role in the metabolism of homocysteine. *MTHFR* reduces the 5,10-methylene tetrahydrofolate to 5-methyltetrahydrofolate and plays the methyl donor role in methionine production from homocysteine [28].

Genome-wide association studies (GWAS) on plasma homocysteine level have reported on the association between the *MTHFR* variant (C667T, rs1801133) and elevated homocysteine levels—therefore the *MTHFR*-encoding gene has been introduced as a genetic determinant of HHcy [29].

Several polymorphisms in the *MTHFR* gene have been found to be associated with various diseases. The *C667T* polymorphism in the *MTHFR* gene (alanine to valine substitution in exon 4), is the most consistently associated with total homocysteine (tHcy), resulting in decreased enzyme activity and elevated homocysteine levels. The *MTHFR A1298C* polymorphism (glutamate to alanine substitution in exon 7) results in a thermos-labile form of enzyme, which may play a role in the regulation of the plasma homocysteine level [30].

Moreover, several polymorphisms have been identified in the 3′-UTR region of the *MTHFR* gene, and are potentially located in the microRNAs (miRNAs) binding sites. The *MTHFR* 2572C > A and 4869C > G variants are located in the 3′-UTR region, and bioinformatics analysis showed that these substitutions create binding sites for several miRNAs, leading to suppression of the translation stage and thus regulating its expression [19].

Factor V is an important protein in the blood coagulation cascade, and its deficiency plays a role in bleeding disorders. *Factor V Leiden* (*G1691A*, rs6025) is a modified form of Factor V leading to activated protein C resistance (APCR) and increased blood clotting. It is introduced as one of the essential genetic risk factors for inherited thrombophilia [31].

In the present study, the frequency of the *MTHFR 677CT* genotype was higher in IS patients than in controls. In addition, the *MTHFR C677T* polymorphism was associated with increased risk of IS in the dominant model. There was no association between the *MTHFR A1298C* and *C2572A* variants and IS. The *MTHFR 4869CG* genotype was associated with lower IS risk, and this polymorphism could be a protective factor in the dominant model. The CCAC haplotype was the most frequent haplotype in both the IS and control groups. Haplotype analysis revealed that the CAAT haplotype of the *MTHFR C4869G*, *C2572A*, *A1298C*, and *C677T* polymorphisms was higher, while the GAAC haplotype was significantly lower in IS patients compared to other haplotypes—and these haplotypes could be considered as risk and protective factors, respectively. There are numerous studies on the relationship between the *MTHFR C677T* and A1298C variants and IS in various ages—and several meta-analyses were performed to assess them. In the meta-analysis performed by Song et al. on 22 case-control studies from January 2000 to October 2014, the *C677T* polymorphism was introduced as a risk factor for IS in dominant, recessive, and allelic models [15]. In another meta-analysis on 40 case-control studies, Chen et al. reported the relation between the *C677T* variant and IS risk in young and middle-aged Asian males [32]. Kumar et al. presented the effect of the *C677T* variant on IS risk in Asians in dominant and recessive models, as well as in Caucasians in recessive but not dominant models [33]. However, Cui showed the association between this polymorphism and adult IS in Asians and Caucasians, but not in Africans [34]. Abhinand et al. analyzed 72 studies and found that this polymorphism may lead to a 1.3-fold increased risk of IS [35].

A meta-analysis by Zhang et al. included 15 studies showing the effect of *A1298C* polymorphism on adult IS, mainly in Asian populations [36]. Sarecka-Hujar et al. performed a meta-analysis to evaluate the relationship between this variant and IS in pediatric patients in eight case-control studies, and found no association between this polymorphism and IS in children—for neither the recessive nor the additive models nor the allelic models [37]. Wei et al. (490 case-control studies) reported the association between *MTHFR A1298C* and *C677T* polymorphisms and IS [38].

Despite the numerous studies and meta-analyses performed on the association between these common polymorphisms and IS, the published reports on the possible effects of variants in the 3′-UTR region of the *MTHFR* gene are few. Recently, Kim et al. analyzed four polymorphisms in the 3′-UTR region of the *MTHFR* gene and IS. Despite the current study results, they found an association between *2572CC* and *6685TT* genotypes and IS in the cardioembolism subgroup. In spite of our findings, they showed no association between *MTHFR* 4869C > G polymorphism and IS [39]. There was an association between *MTHFR* rs142884651 polymorphism in the 3′-UTR region and decreased risk of IS found in a study conducted by Shi et al. They showed that the *MTHFR* 4869C > G variant was associated with increased risk of IS (TC and CC vs. TT), with a poor short-term IS outcome. Indeed, they found elevated serum tHcy levels in *MTHFR* rs868014TC or CC genotypes [40].

In addition, we observed a higher frequency of the *FVL 1691GA* genotype in IS patients, but the difference was not significant. We did not find the prothrombin *20210A* allele in our study population.

Similar to other thrombophilia-related genes, several studies assessed the relationship between *FVL G1691A* and prothrombin *G20210A* polymorphisms and IS in various ethnic groups—and meta-analyses were performed to evaluate the possible effects of these variants on IS susceptibility. In the two separate meta-analyses performed by Hamiz et al. and Bentley et al., prothrombin *G20210A*, *FVL G1691A*, and *MTHFR C677T* polymorphisms were considered as risk factors for IS [41,42]. However, Peck et al. showed a significant protective role of the Factor V Leiden mutation against hemorrhagic stroke [43]. Although Alhazzani et al. reported a significant relationship between Factor V G1691A polymorphism and susceptibility to IS in a dominant model, when they classified the patients in groups according to age, they showed a significant relation between Factor V G1691A polymorphism and IS risk in patients onset at a young age but not at an old age (>40 years) [44].

Some limitations were in the current study that may affect our findings, particularly the relatively low sample size because of the small population in the southeast of Iran. In addition, the different ethnic groups in southeastern Iran and environmental factors could be consider as the other limitations of this investigation. Indeed, if the homocysteine levels in IS patients and controls had been assayed, the findings would have become more appreciated—mainly regarding the possible effects of *MTHFR* polymorphisms on homocysteine levels.

## 5. Conclusions

*MTHFR* C677T polymorphism was associated with increased risk of IS in the dominant model. However, there was no relation between *A1298C* and *C2572A* polymorphisms and IS risk. The *MTHFR* C4869G polymorphism was associated with lower risk of IS in the dominant model. Moreover, for the first time we found that the *CAAT* and *GAAC* haplotypes of *MTHFR* C4869G, *C2572A*, *A1298C*, and *C677T* polymorphisms were associated with higher and lower risk of IS, respectively.

## Figures and Tables

**Table 1:** Demographic characteristics of ischemic stroke (IS) patients and control group.

Variable	Controls n = 157	IS n = 106	*p*-Value
Age (years)	37.2 ±10.8	36.9 ± 10.3	0.8 ^a^
Sex (male/female)	69/88	42/64	0.5 ^b^
Smoking (n, %)	28 (17.8)	29 (27.4)	0.07 ^b^
SBP (mmHg)	117 ± 18	118 ± 24	0.7 ^a^
DBP (mmHg)	75 ± 8.4	77 ± 17	0.3 ^a^
Triglycerides (mg/dL)	100 ± 64	109 ± 79	0.3 ^a^
Total Cholesterol(mg/dL)	148 ± 35	160 ± 39	0.08 ^a^
LDL Cholesterol (mg/dL)	87± 22	92 ± 25	0.1 ^a^
HDL Cholesterol (mg/dL)	39 ± 10	39 ± 11	0.9 ^a^

^a^ Independent *t*-test, the data represent mean ± standard deviation (SD), ^b^ Fisher exact test. SPB: systolic blood pressure, DBP: diastolic blood pressure, LDL: low density lipoprotein, HDL: high density lipoprotein

**Table 2:** Allelic and genotypic frequency of *MTHFR* and *FV* polymorphisms in ischemic stroke patients and the control group.

Polymorphism		IS (n = 106)	Control (n = 157)	*p*-Value	Odds Ratio (95% CI)
***MTHFRC677T*** ***rs1801133***	***genotype***				
	CC, n (%)	66 (62.3)	119 (75.8)		1
	CT, n (%)	32 (30.2)	30 (19.1)	0.028	1.9 (1.1–3.4)
	TT, n (%)	8 (7.5)	8 (5.1)	0.26	1.8 (0.7–5)
	Dominant (CT + TT vs. CC)			0.019	1.9 (1.1–3.3)
	Recessive (TT vs. CT + CC)			0.4	1.5 (0.6–4.2)
	*Allele*				
	C, n (%)	164 (77.4)	268 (85)		1
	T, n (%)	48 (22.6)	46 (15)	0.02	1.7 (1.1–2.7)
***MTHFRA1298C***	***genotype***				
***rs1801131***	AA, n (%)	72 (67.9)	120 (76.4)		1
	AC, n (%)	31 (29.3)	32 (20.4)	0.1	1.6 (0.9–2.9)
	CC, n (%)	3 (2.8)	5 (3.2)	1	1 (0.2–4.3)
	Dominant (AC + CC vs. AA)			0.13	1.5 (0.9–2.7)
	Recessive (CC vs. AC + AA)			0.9	0.9 (0.2–3.8)
	*Allele*				
	A, n (%)	175 (83)	272 (87)		1
	C, n (%)	37 (17)	42 (13)	0.2	1.4 (0.9–2.2)
***MTHFR C2572A*** ***rs4846049***	***genotype***				
	AA, n (%)	25 (23.6)	25 (15.9)		1
	AC, n (%)	50 (47.2)	84 (53.5)	0.12	0.6 (0.3–1.2)
	CC, n (%)	31 (29.2)	48 (30.6)	0.23	0.7 (0.3–1.3)
	Dominant (AC + CC vs. AA)			0.12	0.6 (0.3–1.1)
	Recessive (CC vs. AC + AA)			0.8	0.9 (0.6–1.6)
	*Allele*				
	A, n (%)	100 (47)	134 (43)		
	C, n (%)	112 (53)	180 (57)	0.3	0.8 (0.6–1.2)
***MTHFR C4869G*** ***rs1537514***	***genotype***				
	CC, n (%)	93 (87.7)	117 (74.5)		1
	CG, n (%)	13 (12.3)	38 (24.2)	0.016	0.4 (0.2–0.9)
	GG, n (%)	0	2 (1.3)	1	-
	Dominant (CG + GG vs. CC)			0.01	0.4 (0.2–0.8)
	Recessive (GG vs. CG + CC)			-	-
	*Allele*				
	C, n (%)	199 (94)	272 (87)		
	G, n (%)	13 (6)	42 (13)	0.009	0.4 (0.2-0.8)
***FVL G1691A*** ***rs6025***	***genotype***				
	GG, n (%)	90 (84.9)	144 (91.7)		
	GA, n (%)	16 (15.1)	13 (8.3)	0.09	2 (0.9–4.3)
	AA, n (%)	0 (0)	0 (0)	-	-
	*Allele*				
	G, n (%)	196 (92.5)	301 (96)		
	A, n (%)	16 (7.5)	13 (4)	0.12	1.9 (0.9–4)

**Table 3:** Haplotype analysis of *MTHFR C4869G*, *C2572A*, *A1298C*, and *C677T* polymorphisms in ischemic stroke patients and the control group.

Haplotypes	IS	Control	*p*-Value	OR (95% CI)
CCAC	73 (0.344)	127 (0.404)	0.17	0.8 (0.5–1.1)
CAAC	60 (0.283)	84 (0.268)	0.76	1.1 (0.7–1.6)
GAAC	8 (0.0377)	29 (0.092)	0.02	0.4 (0.2–0.9)
CCCC	16 (0.0750)	16 (0.051)	0.28	1.5 (0.7–3.1)
CAAT	18 (0.0849)	10 (0.032)	0.01	2.8 (1.3–6.2)
CCAT	11 (0.052)	14 (0.045)	0.83	1.2 (0.5–2.6)
CCCT	8 (0.038)	13 (0.041)	0.96	0.9 (0.4–2.2)
CACT	8 (0.0380)	5 (0.016)	0.15	2.4 (0.8–7.5)
GCAC	3 (0.014)	9 (0.029)	0.38	0.5 (0.1–1.8)
CACC	4 (0.019)	3 (0.010)	0.43	2 (0.4–9)

## References

[1] Mukherjee D., Patil C.G. (2011). Epidemiology and the global burden of stroke. World Neurosurg..

[2] Deresse B., Shaweno D. (2015). Epidemiology and in-hospital outcome of stroke in South Ethiopia. J. Neurol. Sci..

[3] Feigin V.L., Forouzanfar M.H., Krishnamurthi R., Mensah G.A., Connor M., Bennett D.A., Moran A.E., Sacco R.L., Anderson L., Truelsen T. (2014). Global and regional burden of stroke during 1990–2010: Findings from the Global Burden of Disease Study 2010. Lancet.

[4] Owolabi M.O., Akarolo-Anthony S., Akinyemi R., Arnett D., Gebregziabher M., Jenkins C., Tiwari H., Arulogun O., Akpalu A., Sarfo F.S. (2015). The burden of stroke in Africa: A glance at the present and a glimpse into the future. Cardiovasc. J. Afr..

[5] (2015). WHO Statistical Information System (WHO SIS): Core Health Indicators Database IRoI.

[6] Caplan L. (2009). Caplan’s Stroke.

[7] Arsene D., Gaina G., Balescu C., Ardeleanu C. (2011). C677T and A1298C methylenetetrahydropholate reductase (MTHFR) polymorphisms as factors involved in ischemic stroke. Rom. J. Morphol. Embryol..

[8] Salimi S., Firoozrai M., Zand H., Nakhaee A., Shafiee S.M., Tavilani H., Mohebbi A. (2010). Endothelial nitric oxide synthase gene Glu298Asp polymorphism in patients with coronary artery disease. Ann. Saudi Med..

[9] Salimi S., Naghavi A., Firoozrai M., Zand H., Tavilani H., Nakhaee A., Mohebbi A. (2012). Association of plasma nitric oxide concentration and endothelial nitric oxide synthase T-786C gene polymorphism in coronary artery disease. Pathophysiology.

[10] Amin F., Jahani M.M., Ghaedi H., Alipoor B., Fatemi A., Tajik M., Sharifi Z., Golmohammadi T., Askari M., Azarnejad A. (2015). Genetic Variants of Cytochrome b-245, Alpha Polypeptide Gene and Premature Acute Myocardial Infarction Risk in an Iranian Population. J. Med. Biochem..

[11] Miranda-Vilela A.L. (2012). Role of polymorphisms in factor V (FV Leiden), prothrombin, plasminogen activator inhibitor type-1 (PAI-1), methylenetetrahydrofolate reductase (MTHFR) and cystathionine beta-synthase (CBS) genes as risk factors for thrombophilias. Mini Rev. Med. Chem..

[12] Hoffbrand A.V.M.P., Pettit J.A. (2006). Thrombosis and Antithrombotic Therapy. Essential Haematology.

[13] D’Uva M., Micco P.D., Strina I., Placido G.D. (2010). Recurrent pregnancy loss and thrombophilia. J. Clin. Med. Res..

[14] Santilli F., Davi G., Patrono C. (2016). Homocysteine, methylenetetrahydrofolate reductase, folate status and atherothrombosis: A mechanistic and clinical perspective. Vascul. Pharmacol..

[15] Song Y., Li B., Wang C., Wang P., Gao X., Liu G. (2016). Association between 5,10-Methylenetetrahydrofolate Reductase C677T Gene Polymorphism and Risk of Ischemic Stroke: A Meta-analysis. J. Stroke Cerebrovasc. Dis..

[16] Carp H., Salomon O., Seidman D., Dardik R., Rosenberg N., Inbal A. (2002). Prevalence of genetic markers for thrombophilia in recurrent pregnancy loss. Hum Reprod..

[17] Holmes M.V., Newcombe P., Hubacek J.A., Sofat R., Ricketts S.L., Cooper J., Breteler M.M., Bautista L.E., Sharma P., Whittaker J.C. (2011). Effect modification by population dietary folate on the association between MTHFR genotype, homocysteine, and stroke risk: A meta-analysis of genetic studies and randomised trials. Lancet.

[18] Mohammadpour-Gharehbagh A., Teimoori B., Narooei-Nejad M., Mehrabani M., Saravani R., Salimi S. (2018). The association of the placental MTHFR 3’-UTR polymorphisms, promoter methylation, and MTHFR expression with preeclampsia. J. Cell Biochem..

[19] Mohammadpour-Gharehbagh A., Salimi S., Keshavarzi F., Saeidian F., Mousavi M., Teimoori B., Esmaeilipour M., Mokhtari M. (2018). Genetic variants in 3’-UTRs of MTHFR in the pregnancies complicated with preeclampsia and bioinformatics analysis. J. Cell Biochem..

[20] Salimi S., Saravani M., Yaghmaei M., Fazlali Z., Mokhtari M., Naghavi A., Farajian-Mashhadi F. (2015). The early-onset preeclampsia is associated with MTHFR and FVL polymorphisms. Arch Gynecol. Obstet..

[21] Ueland P.M., Refsum H., Beresford S.A., Vollset S.E. (2000). The controversy over homocysteine and cardiovascular risk. Am. J. Clin. Nutr..

[22] Austin R.C., Lentz S.R., Werstuck G.H. (2004). Role of hyperhomocysteinemia in endothelial dysfunction and atherothrombotic disease. Cell Death Differ..

[23] Buemi M., Marino D., Di Pasquale G., Floccari F., Ruello A., Aloisi C., Corica F., Senatore M., Romeo A., Frisina N. (2001). Effects of homocysteine on proliferation, necrosis, and apoptosis of vascular smooth muscle cells in culture and influence of folic acid. Thromb Res..

[24] White W.M., Turner S.T., Bailey K.R., Mosley T.H., Jr Kardia S.L., Wiste H.J., Kullo I.J., Garovic V.D. (2013). Hypertension in pregnancy is associated with elevated homocysteine levels later in life. Am. J. Obstet. Gynecol..

[25] Schaffer A., Verdoia M., Cassetti E., Marino P., Suryapranata H., De Luca G., Novara Atherosclerosis Study G. (2014). Relationship between homocysteine and coronary artery disease. Results from a large prospective cohort study. Thromb Res..

[26] Lehotsky J., Tothova B., Kovalska M., Dobrota D., Benova A., Kalenska D., Kaplan P. (2016). Role of Homocysteine in the Ischemic Stroke and Development of Ischemic Tolerance. Front Neurosci..

[27] Kullo I.J., Ding K., Boerwinkle E., Turner S.T., Mosley T.H., Kardia S.L., de Andrade M. (2006). Novel genomic loci influencing plasma homocysteine levels. Stroke.

[28] Trimmer E.E. (2013). Methylenetetrahydrofolate reductase: Biochemical characterization and medical significance. Curr. Pharm. Des..

[29] Van Meurs J.B., Pare G., Schwartz S.M., Hazra A., Tanaka T., Vermeulen S.H., Cotlarciuc I., Yuan X., Malarstig A., Bandinelli S. (2013). Common genetic loci influencing plasma homocysteine concentrations and their effect on risk of coronary artery disease. Am. J. Clin. Nutr..

[30] Weisberg I.S., Jacques P.F., Selhub J., Bostom A.G., Chen Z., Curtis Ellison R., Eckfeldt J.H., Rozen R. (2001). The 1298A-->C polymorphism in methylenetetrahydrofolate reductase (MTHFR): In vitro expression and association with homocysteine. Atherosclerosis.

[31] Kujovich J.L. (2011). Factor V Leiden thrombophilia. Genet. Med..

[32] Chen M., Mao B.Y., Wang D., Cheng X., Xu C.X. (2016). Association between rs1801133 polymorphism and risk of adult ischemic stroke: Meta-analysis based on case-control studies. Thromb Res..

[33] Kumar A., Kumar P., Prasad M., Sagar R., Yadav A.K., Pandit A.K., Jali V.P., Pathak A. (2015). Association of C677T polymorphism in the methylenetetrahydrofolate reductase gene (MTHFR gene) with ischemic stroke: A meta-analysis. Neurol Res..

[34] Cui T. (2016). MTHFR C677T mutation increased the risk of Ischemic Stroke, especially in large-artery atherosclerosis in adults: An updated meta-analysis from 38 researches. Int. J. Neurosci..

[35] Abhinand P.A., Manikandan M., Mahalakshmi R., Ragunath P.K. (2017). Meta-analysis study to evaluate the association of MTHFR C677T polymorphism with risk of ischemic stroke. Bioinformation.

[36] Zhang M.J., Hu Z.C., Yin Y.W., Li B.H., Liu Y., Liao S.Q., Gao C.Y., Li J.C., Zhang L.L. (2014). A meta-analysis of the relationship between MTHFR gene A1298C polymorphism and the risk of adult stroke. Cerebrovasc. Dis..

[37] Sarecka-Hujar B., Kopyta I., Skrzypek M. (2018). Is the 1298A>C polymorphism in the MTHFR gene a risk factor for arterial ischaemic stroke in children? The results of meta-analysis. Clin. Exp. Med..

[38] Wei L.K., Au A., Menon S., Griffiths L.R., Kooi C.W., Irene L., Zhao J., Lee C., Alekseevna A.M., Hassan M.R.A. (2017). Polymorphisms of MTHFR, eNOS, ACE, AGT, ApoE, PON1, PDE4D, and Ischemic Stroke: Meta-Analysis. J. Stroke Cerebrovasc. Dis..

[39] Kim J.O., Park H.S., Ryu C.S., Shin J.W., Kim J., Oh S.H., Kim O.J., Kim N.K. (2017). Interplay between 3’-UTR polymorphisms in the methylenetetrahydrofolate reductase (MTHFR) gene and the risk of ischemic stroke. Sci. Rep..

[40] Shi J., He W., Wang Y., Hua J. (2018). Tagging Functional Polymorphism in 3’ Untranslated Region of Methylene Tetrahydrofolate Reductase and Risk of Ischemic Stroke. Cell Physiol. Biochem..

[41] Bentley P., Peck G., Smeeth L., Whittaker J., Sharma P. (2010). Causal relationship of susceptibility genes to ischemic stroke: Comparison to ischemic heart disease and biochemical determinants. PLoS ONE.

[42] Hamzi K., Tazzite A., Nadifi S. (2011). Large-scale meta-analysis of genetic studies in ischemic stroke: Five genes involving 152,797 individuals. Indian J. Hum. Genet..

[43] Peck G., Smeeth L., Whittaker J., Casas J.P., Hingorani A., Sharma P. (2008). The genetics of primary haemorrhagic stroke, subarachnoid haemorrhage and ruptured intracranial aneurysms in adults. PLoS ONE.

[44] Alhazzani A.A., Kumar A., Selim M. (2018). Association between Factor V Gene Polymorphism and Risk of Ischemic Stroke: An Updated Meta-Analysis. J. Stroke Cerebrovasc. Dis..

